# Mechanical Properties and Microstructure of a NiCrFeCoMn High-Entropy Alloy Deformed at High Strain Rates

**DOI:** 10.3390/e20110892

**Published:** 2018-11-21

**Authors:** Bingfeng Wang, Xianrui Yao, Chu Wang, Xiaoyong Zhang, Xiaoxia Huang

**Affiliations:** 1 State Key Laboratory for Powder Metallurgy, Central South University, Changsha 410083, China; 2 School of Materials Science and Engineering, Central South University, Changsha 410083, China

**Keywords:** high-entropy alloy, electron microscopy, plasticity methods, plasticity, serration behavior

## Abstract

The equiatomic NiCrFeCoMn high-entropy alloy prepared by arc melting has a single crystallographic structure. Mechanical properties and microstructure of the NiCrFeCoMn high-entropy alloy deformed at high strain rates (900 s^−1^ to 4600 s^−1^) were investigated. The yield strength of the NiCrFeCoMn high-entropy alloy is sensitive to the change of high strain rates. Serration behaviors were also observed on the flow stress curves of the alloy deformed at the strain rates ranging from 900 s^−1^ to 4600 s^−1^. The Zerilli–Armstrong constitutive equation can be used to predict the flow stress curves of the NiCrFeCoMn high-entropy alloy. Large amounts of deformation bands led to obvious serration behaviors of the NiCrFeCoMn high-entropy alloy under dynamic loading.

## 1. Introduction

The concept of multi-component high-entropy alloys (HEAs) has been presented in the beginning of this century. Most HEAs usually contain five elements with nearly equal atomic ratios [1]. HEAs have excellent mechanical properties, such as low strength and high plasticity [2,3,4,5,6,7,8,9,10,11,12]. From recent literatures, the hardness of FeCoNiCrMn high-entropy alloy has even increased to 6700 MPa [13]. The Al_0.6_CoCrFeNi high-entropy alloy also displayed the excellent strength–ductility combination for nanoscale deformation twins induced by dynamic loading and high-density dislocation substructure [14]. However, the NiCrFeCoMn high-entropy alloy has still been regarded as a typical case for its single-phase face-centered cubic (FCC) [15] and relative promising mechanical properties. For example, its yield strength increases with the angle of rotation at high-pressure torsion (HPT) at room temperature at the cost of reduced ductility [16] and can reach up to 350 MPa when reducing its grain size [17,18]. Therefore, the NiCrFeCoMn high-entropy alloy prepared by arc melting can be used to fabricate big parts of industry devices or as the transitional layer between the two types of alloys, e.g., the HEA solder used for welding pure titanium and chromium–nickel–titanium stainless steel [19]. At the present time, dynamic impacts are widely found in aeronautical engineering, the automotive industry, and marine engineering. The as-cast NiCrFeCoMn high-entropy alloy could be applied to many dynamic deformation processes such as penetration, impact cyclic loading, and shock loading. Hence, it is vital to comprehend the dynamic behavior of as-cast NiCrFeCoMn high-entropy alloy under low/high-speed loading and expand its applications. 

Many researchers were devoted to investigating the deformation behavior of the high-entropy alloy at different strain rate levels (1 × 10^−8^ s^−1^ to 10 s^−1^) [20]. From the recent research, serration behavior was found in the high-entropy alloy CoCrFeMnNi prepared by powder metallurgy at a low strain rate of 1 × 10^−3^ s^−1^ and the high strain rates (1 × 10^3^ s^−1^ to 3 × 10^3^ s^−1^) [21]. Serration behavior was supposed to be associated with Cottrell atmosphere interaction with moving dislocations, slip bands, and dynamic strain aging, i.e., the dynamic breakaway/locking of dislocations from/by mobile solute atoms at intermediate temperatures. Until now, only several researches on dynamic behavior of high-entropy alloys under high strain rates (beyond 1 × 10^3^ s^−1^) have been published. Kumar et al. [22] had investigated the strain-rate sensitivity of yield strength for the Al_0.1_CrFeCoNi high-entropy alloy under high strain rates. Li et al. [23] had also suggested that the Al_0.3_CoCrFeNi high-entropy alloy exhibits high strain-rate sensitivity. Dirras et al. [24] had found that the Ti_20_Hf_20_Zr_20_Ta_20_Nb_20_ high-entropy alloy would be strongly localized under deformation at high strain rates. He et al. [25] had discussed the strain-rate sensitivity effect for the FeCoNiCrMn high-entropy alloy and found a higher strain-rate sensitivity of 0.022 than that of traditional FCC metals. Park et al. [26] had even found that the strain-rate dependency of the yield strength under dynamic conditions would be much higher than that under quasi-static conditions. However, the mechanism of the special phenomenon for the high-entropy alloy deformed at high strain rates (beyond 1 × 10^3^ s^−1^) is not clear yet. Serration characteristics are ubiquitous in many structural and functional materials such as high-entropy alloy (AlCoCr_1.5_Fe_1.5_NiTi_0.5_, Al_0.3_CoCrFeNi etc.). It would lead to the instability of mechanical properties. By studying the serration behavior, it may be possible to have an early warning signal for oncoming epileptic seizures and perhaps economic trends.

In the present work, we used the split-Hopkinson pressure bar to investigate the dynamic mechanical behavior of NiCrFeCoMn high-entropy alloy prepared by arc melting. The aims are: (1) To report the mechanical properties and microstructure under dynamic loadings, (2) to obtain the plastic model, and (3) to discuss the microstructural mechanism for the serration behavior when the alloy deforms at high strain rates.

## 2. Experiments and Procedures

**Figure 1:**
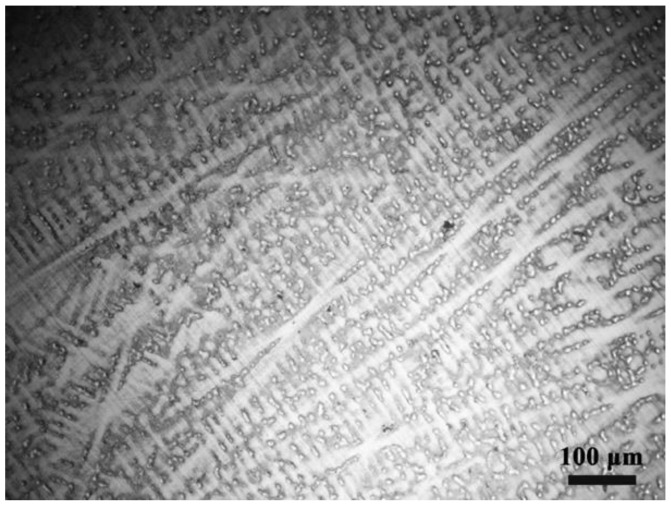
Optical micrograph of the as-cast NiCrFeCoMn alloy.

**Figure 2:**
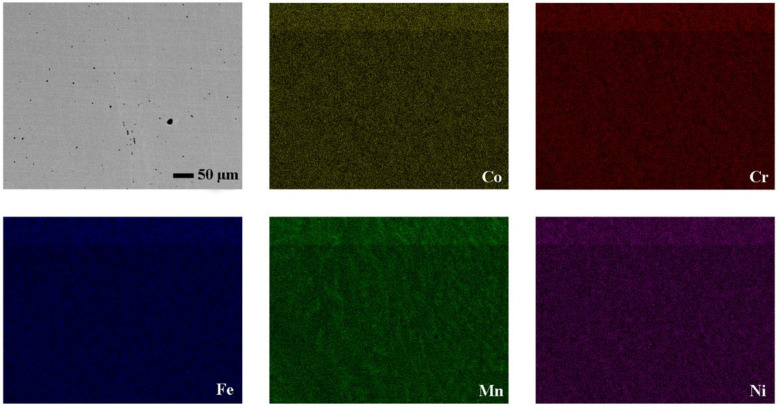
Element planar pattern of the as-cast NiCrFeCoMn alloy.

**Figure 3:**
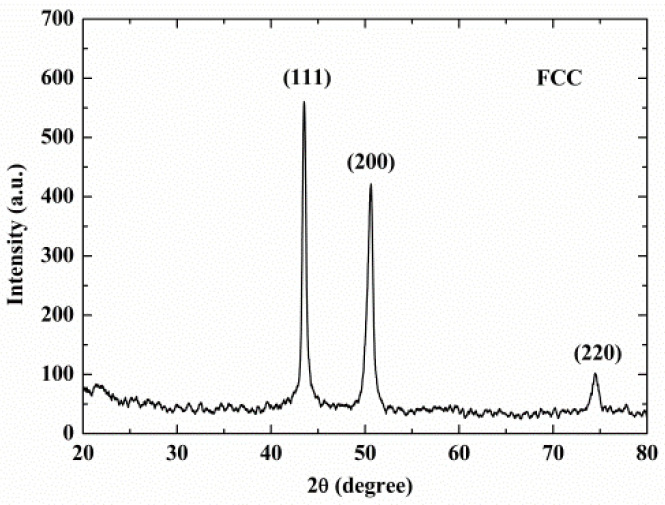
The XRD pattern of the as-cast NiCrFeCoMn alloy.

The material was prepared by arc melting and the chemical composition is given in Table 1. Figure 1 shows the optical micrograph of the initial microstructure of the sample. The obtained specimen exhibits an as-cast dendrite structure and the columnar crystals are distributed evenly. Elemental scanning results in Figure 2 show the distribution of each element evenly in the alloy. From the XRD pattern shown in Figure 3, it can be found that the NiCrFeCoMn high-entropy alloy is composed of a simple FCC solid solution.

In this investigation, cylindrical specimens were used for mechanical testing. To ensure uniaxial compressive condition, the end faces of the compressive specimens were ground on each side with SiC paper. During the process of the impact loading and the electrical signal collection, we had adopted advanced waving plastic and anti-jamming techniques. Two kinds of compressive tests were adopted to do mechanical testing at an ambient temperature (298 K) as follows: (1) Quasi-static compressive tests were performed with an INSTRON 3369 machine at the strain rate of 1 × 10^−3^ s^−1^, (2) dynamic compressive tests were performed with a split-Hopkinson pressure bar at the strain rates of 900 s^−1^, 1700 s^−1^, and 4600 s^−1^. The cylindrical specimens had three diameters. Specimens for the strain rate of about 900 s^−1^ had a height of 8.4 mm and diameter of 6 mm, for the strain rate of about 1700 s^−1^ they had a height of 5.6 mm and diameter of 4 mm, and for the strain rate of about 4600 s^−1^ they had a height of 2.8 mm and diameter of 2 mm. The strain rate, the true strain, and the true stress of cylindrical specimens can be obtained by the following equations:

(1) $$\dot{\varepsilon }=-\frac{2C_{0}}{L_{S}}\varepsilon_{r}\left(t\right),$$

(2) $$\varepsilon =-\frac{2C_{0}}{L_{S}}\int_{0}^{t}\varepsilon_{r}\left(t\right)dt,$$

(3) $$\varepsilon =-\frac{2C_{0}}{L_{S}}\int_{0}^{t}\varepsilon_{r}\left(t\right)dt,$$

where E_0_ and C_0_ are elastic modulus and elastic wave speed in a split-Hopkinson pressure bar, A_0_ is the cross-sectional area of the bar, A_s_ and L_s_ are the cross-sectional area and the length of the cylindrical specimens, and *ε_r_*(*t*) and *ε_t_*(*t*) are the experimentally measured strain of incident and transmitted stress pulse on the split-Hopkinson pressure bars respectively.

Cylindrical specimens were sectioned to two halves along the impacting axis by line cutting. Afterwards, the half sections were polished with the etchant of 50 mL hydrochloric acid, 50 mL water, and 10 g CuSO_4_·5H_2_O. Samples were cured and ground using SiC papers. Polishing steps were employed by using diamond pastes and polishing cloths. A mixture solution of 90% acetic acid and 10% perchloric acid at room temperature and at an applied voltage of 27 V for 15 s was then used for electro-polishing. Optical microscopy was performed with a POLYVAR-MET microscope. The crystallographic structure was identified by X-ray diffraction (Rigaku D/MAX-2500 X-ray diffractometer, Rigaku Corporation, Tokyo, Japan) using a Cu target at an operating voltage of 40 kV and current of 250 mA. Electron backscatter diffraction (EBSD) patterns were collected using a ZEISS EVOMA10 scanning electron microscope (SEM, Carl Zeiss SMT Ltd., Cambridge, UK) equipped with a detector and operated at an accelerating voltage of 20 kV. The working distance for SEM is about 10 mm. Transmission electron microscopy (TEM, Royal Philips, Amsterdam, Netherlands) observations were carried out with a Tecnai G2 T20 ST transmission electron microscope operated at 200 kV.

## 3. Results

**Figure 4:**
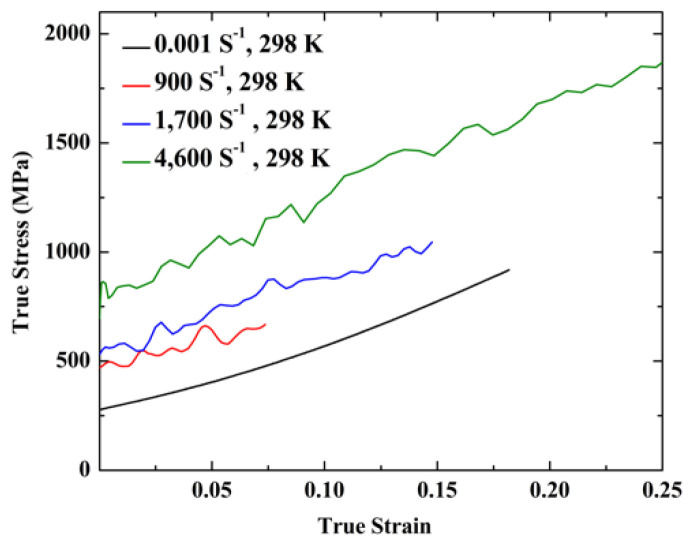
Compressive true stress–strain curves of the NiCrFeCoMn high-entropy alloy at different strain rates.

Figure 4 presents the true stress–strain curves of the NiCrFeCoMn high-entropy alloy. It can be seen that the yield strength of the NiCrFeCoMn high-entropy alloy increased from 490 MPa to 800 MPa, with the strain rates varying from 900 s^−1^ to 4600 s^−1^. The flow stress curves are likely smoothed when the specimens are deformed at the strain rate of 0.001 s^−1^ at an ambient temperature (298 K). However, the serrations appeared on the flow stress curves of the specimens deformed at high strain rates (e.g., 900 s^−1^, 1700 s^−1^, and 4600 s^−1^). Furthermore, with the increasing of the strain rates, the serrations on the flow stress curves became more serious. 

**Figure 5:**
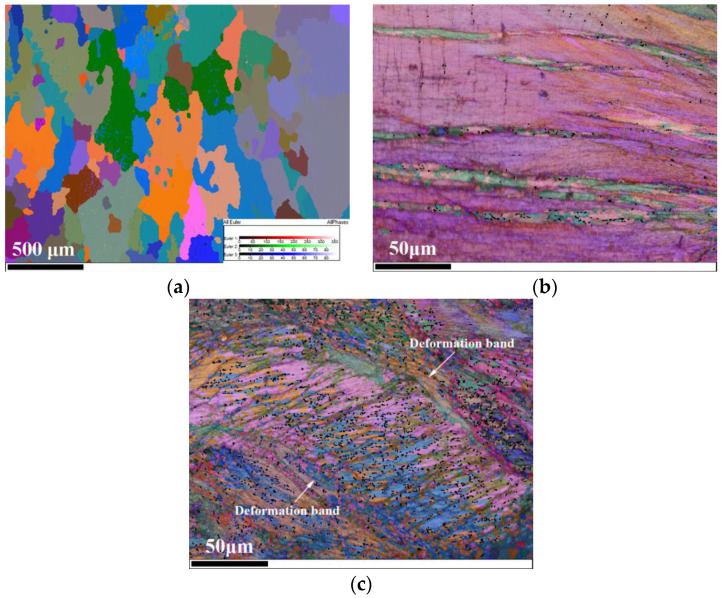
Electron backscattered diffraction images. (**a**) the Euler image of the as-received specimen; (**b**) the Euler+BC image of the deformed specimen at the strain rate 1 × 10^−3^ s^−1^; (**c**) the Euler+BC image of the deformed specimen at the strain rate 4600 s^−1^.

The EBSD technique was used to investigate the microstructure and the micro-orientation of a NiCrFeCoMn high-entropy alloy. Figure 5a–c are the electron backscattered diffraction images of the as-received specimen, the specimen deformed at the strain rate of 0.001 s^−1^, and the specimen deformed at the strain rate of 4600 s^−1^. First, the noise of the images was reduced. Then, to Figure 5b,c were added a layer of full Euler angles after adding a layer of band contrast. The size of the Kuwahara filter was 3-pixel points × 3-pixel points and the smoothing angle was 5°. Among them, high-angle boundaries (60°) were marked with black lines. It is evident that the alloy in the as-cast condition consisted of grains on the order of micrometers in size. A visual comparison of Figure 5b,c suggests a higher density of high-angle boundaries in the specimen deformed at a high strain rate of 4600 s^−1^ compared to the specimen deformed at a low strain rate of 0.001 s^−1^. Further, the intensive deformation bands were generated in the specimen deformed at a high strain rate of 4600 s^−^^1^, as shown in Figure 5c. Therefore, the obvious serrations in flow stress curves of the specimen deformed at high strain rates are caused by the intensive deformation bands.

**Figure 6:**
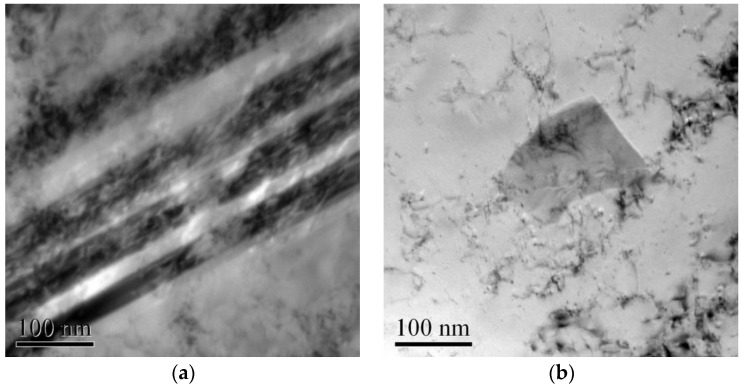

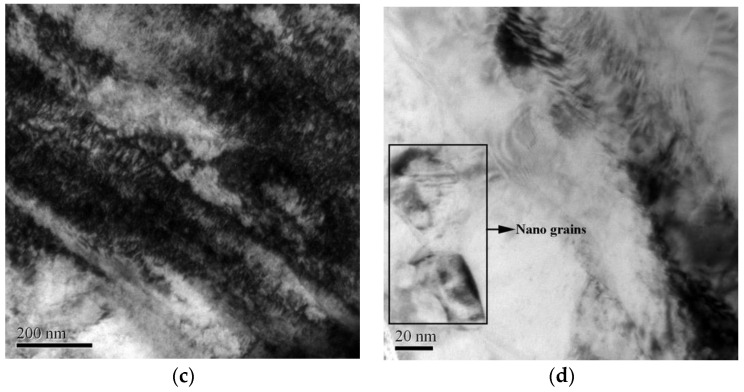
Bright field electron images showing microstructure in the specimens deformed at high strain rates. (**a**) and (**b**) are for the specimen deformed at the strain rate of about 1700 s^−1^ (**c**) and (**d**) are for the specimen deformed at the strain rate of about 4600 s^−1^.

Bright field electron images taken for the specimens deformed at strain rates of 1700 s^−1^ and 4600 s^−1^ are shown in Figure 6. Figure 6a,c show the high-density dislocations and deformation bands that were formed in the NiCrFeCoMn high-entropy alloy. These deformation bands were distributed in parallel. The deformation band consisted of nanograins, as shown in Figure 6b,d. Comparing Figure 6a,b and Figure 6c,d, the parallel deformation bands were more clear when the specimen was deformed at relative lower strain rates as shown in Figure 6a,c, and the sizes of the nanograins in the specimens under a strain rate of 1700 s^−1^ were larger than those in the specimens deformed at a strain rate of 4600 s^−1^. 

## 4. Discussion

### 4.1. The Constitutive Model and Strain-Rate Sensitivity

The main dynamic constitutive equations are the Johnson–Cook and Zerilli–Armstrong plastic models. Among them, the Johnson–Cook model is the most widely used for its relative simple expression. It can be represented as follows [27,28]:

(4) $$\sigma =\left(A+B\varepsilon^{n}\right)\left[1+C\ln\left(\frac{\dot{\varepsilon }}{\dot{\varepsilon_{0}}}\right)\right]\left[1-{\left(\frac{T-T_{r}}{T_{m}-T_{r}}\right)}^{m}\right],$$

where A, B, and C are material constants, σ and ε are the flow stress and the equivalent plastic strain respectively, $\dot{\varepsilon }$ and *T* are the equivalent plastic strain rate and the experimental temperature respectively, and *T_r_* and *T_m_* are the reference temperature (usually room temperature) and the melting point, respectively.

The dislocation mechanism is important for studying the plastic deformation of metallic materials under dynamic deformation. Therefore, the Zerilli–Armstrong plastic model improves the Johnson–Cook model on the basis of dislocation mechanism [29]. In this work, the Zerilli–Armstrong plastic model was used for predicting the strain rate flow behavior of the as-cast NiCrFeCoMn high-entropy alloy. It can be represented as follows:

(5) $$\sigma =C_{0}+C_{1}\times \varepsilon^{P}\times exp\left[-C_{2}T+C_{3}T\ln\left(\frac{\dot{\varepsilon }}{\dot{\varepsilon_{0}}}\right)\right],$$

where C_0_, C_1_, C_2_, C_3_, and P are material constants. Note that T is 298 K. 

Taking initial values of the Johnson–Cook model as follows: A = 1, B = 4, C = 100, *n* = 1, then the parameter values and the constitutive relation of stress-strain with strain rate could be obtained by using the MATLAB program (Version 7.0). Therefore, the constitutive equation based on the Johnson–Cook plastic model can be obtained as follows:

(6) $$\sigma =\left(0.6039+4.81\varepsilon^{1}\right)\left[1+121.6\ln\left(\frac{\dot{\varepsilon }}{\dot{\varepsilon_{0}}}\right)\right].$$

As above, taking initial values of the Zerilli–Armstrong model as follows: C_0_ = 400, C_1_ = 9 × 10^5^, C_2_ = 0.01, C_3_ = 0.001, P = 0.5, then the constitutive equation based on the Zerilli-Armstrong plastic model can be obtained as follows:

(7) $$\sigma =473.3+9.3\times 10^{5}\times \varepsilon^{0.5}\times exp\left[-0.0415T+0.0026T\ln\left(\frac{\dot{\varepsilon }}{\dot{\varepsilon_{0}}}\right)\right].$$

**Figure 7:**
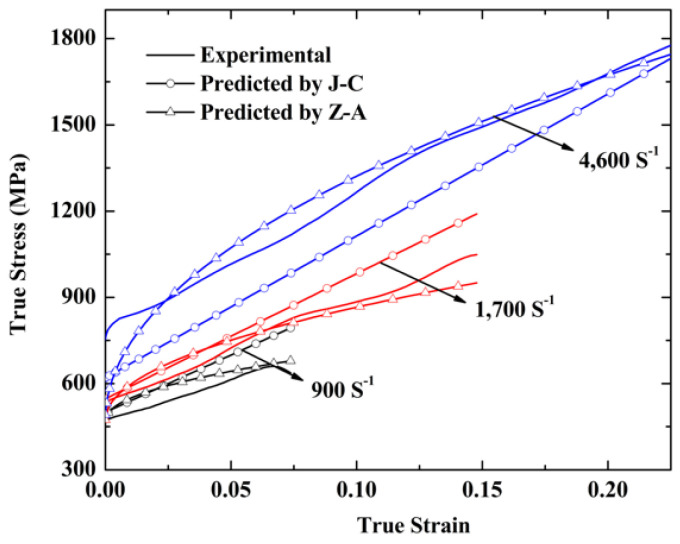
Comparison between the experimental results and the stress calculated by the Johnson–Cook and the Zerilli–Armstrong plastic models at 298K.

Figure 7 shows the comparison of the calculated results obtained from Equations (6) and (7) and the experimental data of the NiCrFeCoMn high-entropy alloy specimens. It can be seen that the results predicted by the Zerilli–Armstrong plastic model are in better agreement with the experimental results.

**Figure 8:**
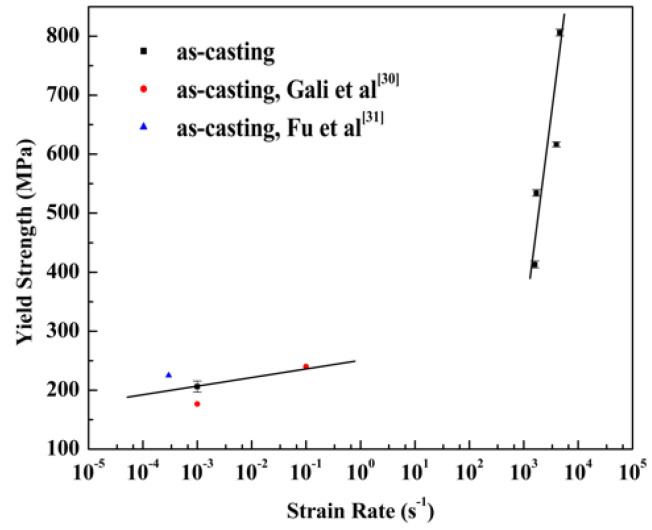
The yield strength vs. strain rate curves of the NiCrFeCoMn high-entropy alloy [30,31].

Figure 8 shows the yield strength vs. strain rate curves of the as-cast NiCrFeCoMn high-entropy alloy as a function of strain rate at ambient temperature. The strain-rate sensitivity is defined as follows:

(8) $$m=\frac{d\left(\log\sigma \right)}{d\left(\log\dot{\varepsilon }\right)}.$$

Notice that the yield strength distributes from 200 MPa to 800 MPa and the slope of the tangent for the curve is increasing with the increase of strain rates. Therefore, the as-cast NiCrFeCoMn high-entropy alloy has distinguished strain-rate sensitivity at high strain rates.

He et al. [32] studied the stress exponent (the reciprocal of strain rate sensitivity) of the NiCrFeCoMn high-entropy alloy deformed under strain rates less than 10^−2^ s^−1^. He found that the stress exponent was in a positive relationship with the strain rates. When the strain rate ranged from 3.205 × 10^−5^ to 8.013 × 10^−4^ s^−1^, the stress exponent increased simultaneously. Moon et al. [33] also studied the strain rate sensitivity at the elevated temperature and the cryogenic temperature, respectively. The results showed that the flow stress at 77 K was higher than that at room temperature. Therefore, the strain rate sensitivity of the flow stress at RT was higher than that at 77 K under strain rates less than 1 × 10^−2^ s^−1^. The following formula can explain this phenomenon:

(9) $$V^{*}=\sqrt{3}kT\frac{\partial \ln\dot{\varepsilon }}{\partial \sigma },$$

where k is the Boltzmann constant and V* is activation volume.

The NiCrFeCoMn high-entropy alloy had positive activation volume at strain rates less than 1 × 10^−2^ s^−1^. However, when the specimen deformed under high strain rates (beyond 1 × 10^3^ s^−1^), the deformation time was very short. There was not enough time available for thermal energy to help dislocations overcome the barriers. The NiCrFeCoMn high-entropy alloy had a different deformation mechanism at dynamic loading. According to the literature [22], the large jump in yield strength at high strain rates is probably due to the phonon drag effect on the motion of dislocations. The phonon drag phenomenon becomes very effective during plastic deformation at high strain rates [22]. A phonon is an elastic lattice vibration propagating in a crystal [26]. The viscous drag generating from the interaction of the dislocations has negative impacts on the deformation progress. The drag effects by the phonons can be ignored under quasi-static conditions for low dislocation velocities. However, high dislocation velocities under high strain rates would enhance the phonon drag effects greatly and lead to phonons scattering, which also hinders dislocation movement. Therefore, the dynamic deformations lead to a much higher strain rate dependence of the flow stress than those under quasi-static conditions.

### 4.2. Mechanism for the Serration Behavior

High-entropy alloys present serrations on the flow stress curves during the plastic deformation, often at a normal strain rate around 1 × 10^−4^ s^−1^ or at low temperatures. Serration behavior exhibits in the stress-strain curves of the Al_0.5_CoCrCuFeNi high-entropy alloy at 7 K, 7.5 K, and 9 K at a strain rate of 4 × 10^−4^ s^−1^ [19]. The Al_5_Cr_12_Fe_35_Mn_28_Ni_20_ high-entropy alloy exhibits typical serration behaviors at the elevated temperatures of 573 K and 673 K, with a strain rate of 1 × 10^−4^ s^−1^ [34]. Several microstructure mechanisms are proposed to explain the serration behavior, e.g., the Portevin-Le Chatelier (PLC) effect. Serration behavior was supposed to be associated with Cottrell atmosphere interaction with moving dislocations, slip bands, and dynamic strain aging. In Figure 4, it can be seen that the stress-strain curves of the as-cast NiCrFeCoMn high-entropy alloy deformed at a strain rate of 1 × 10^−3^ s^−1^ show no serrations, and marked serrations are present on the stress-strain curves of specimens deformed at strain rates above 900 s^−1^. With increasing strain rates, amplitudes of the serrations on the stress-strain curves become much larger. Investigations on the microstructure in the as-cast NiCrFeCoMn high-entropy alloy show that the high density dislocations and the deformation bands are generated in the specimens deformed at high strain rates, shown in Figure 5 and Figure 6. The Portevin-Le Chatelier effect, i.e., Cottrell atmosphere interaction with moving a simple dislocation structure, may not be the main reason for the serration behavior of the as-cast NiCrFeCoMn high-entropy alloy becoming deformed at dynamic loadings. On the other hand, if the dynamic deformation becomes more serious and the value of the strain rate increases, deformation bands are generated in the as-cast NiCrFeCoMn high-entropy alloy and the amplitudes of the serrations on the stress-strain curves become much larger. Therefore, large amounts of the deformation bands, leading to the serration behaviors, play an important role for the mechanical properties of the high-entropy alloy.

## 5. Conclusions

The as-cast equiatomic NiCrFeCoMn high-entropy alloy has a simple FCC crystallographic structure. Serration behavior is observed in the stress- strain curves of the as-cast NiCrFeCoMn high-entropy alloy deformed at dynamic loadings. The yield strength of the high-entropy alloy, which distributes from 490 to 800 MPa, presents a positive relationship with the strain rates. The Johnson–Cook plastic model and the Zerilli–Armstrong plastic model of the NiCrFeCoMn high-entropy alloy are obtained. However, the results predicted by Zerilli–Armstrong correspond better with the experimental results. The serration behavior of the NiCrFeCoMn high-entropy alloy at a high strain rate is sensitive to the strain rates. The high density of deformation bands plays an important role in the deformation behavior and mechanical properties of the as-cast NiCrFeCoMn high-entropy alloy deformed at dynamic loadings.

## Figures and Tables

**Table 1:** Chemical composition of the NiCrFeCoMn high-entropy alloy.

Elements	Cr	Mn	Fe	Co	Ni
wt.%	16.42	18.06	21.64	21.71	22.18
at.%	17.76	18.49	21.79	20.72	21.24
